# Veterinary Medical Register

**Published:** 1881-10

**Authors:** 


THE VETERINARY MEDICAL REGISTER, which we published in our last number has been received with much favor. It has, we believe, done a great deal to make veterinarians known to each other, and has tended to stimulate the cause of veterinary education. We have in hand a considerable number of additional names. It seems better, however, to wait until the next issue before publishing them. By that time we shall doubtless be able to make our list very complete. We again urge all veterinarians whose names are not recorded to forward the ma tonce.

